# Infidelity and Contempt of Religion

**Published:** 1880-06

**Authors:** 


INFIDELITY AND CONTEMPT OF RELIGION seem to have taken root, we are happy to say, in only a few educational institutions in this country at the present time; still their baleful and destructive fruit is scattered by those who receive its pernicious seeds, at the hands of their teachers, as they go forth on the business of after life. Parents and guardians should therefore exercise the greatest caution in the selection of the schools their children attend so as to avoid the evil ones. This admonition is particularly necessary in regard to a medical school. "By faith are ye saved," is the language of Holy writ; and the words need no comment from mortal lip or pen, to prove their equal application to the people of our day. The heartless skeptic and scoffing infidel are less to be feared in their public harangues, however rhetorical and beautiful the language in which their baneful arrogance may be clothed, than the "Freethinker" who sustains the relation of preceptor to the young.

The teacher may be commonplace and even prosy in his prelections before his class, but if not actually discourteous in his address, his words will be remembered, and reverted to in future years, for guidance and direction to the thoughts or actions of his pupils. How necessary then, is it, to see that the youth of the present day, when atheism and infidelity are abroad in the land, be sent to schools where *Christianity* is not only *a recognized principle of faith*, but where the *professors* and *teachers practice and respect* the doctrines of the lowly Nazarine.

Should the "true inwardness" of a certain faculty become known to the mothers and matrons of this country, as it has been revealed to us, we do not hesitate to say that *immediately thereafter*, those "professors" would harangue to empty benches, or only to those who are without faith, and without God in the world. There is a beautiful narrative of the East that gives to mortals one visible guardian angel that ever watches over them, and points upward! And why may not this Oriental legend be a bright allegorical symbol of the ceaseless and undying love of a mother that leaps into existence on the advent of her innocent offspring? When the christian mother beholds the dawn that is to usher in the bright intellectual day of her little one, and bending its tender knees, instinctively points its eyes to Calvary and its blood stained cross, and anon, tells him of life, and of death, of a judgment, and an eternity to come; she tries to assure him that *faith* in the Crucified Redeemer will certainly save the soul of her dear one. When this lesson has been learned, though all earthly joy should decay, and hope flee as a phantom away; salvation is assured, and comfort felt, that He who redeemed him will never leave nor forsake him but shield and save him in the final hour. Curses and anathemas more dire and deep, if possible, should rest upon the *inhuman* wretch who would attempt with sophistry or vulgar jest, to plant the seed of *unbelief* in such a young heart as this! More direful indeed, than those Queen Anne invoked upon the monster, Duke of Gloucester, should be his reward. We cannot better conclude this brief but most important matter, than in the language of Sir H. Davy: "I envy no quality of the mind or intellect of others—no genius, wit, nor fancy; but if I should choose what would be most delightful, and I believe, most useful to me, I prefer a *firm religious belief to any other blessing*, for it makes discipline of good, creates new hopes when earthly hopes vanish, and throws over the decay, the destruction of existence, the most gorgeous of all lights; awakens life in death, and from corruption and decay, calls up beauty and divinity; makes an instrument of misfortune and of shame the ladder of ascent to paradise, and far above all combinations of earthly hopes, calls up the most delightful visions of palms and amaranths—the gardens of the blest, and the security of everlasting joys, where the sensualist and the skeptic view only gloom, decay, annihilation and despair."

HARRY TIMROD.—Just as we were concluding our editorial remarks for this number of the journal, a little brochure with the sad and simple cognomen Timrod, came into our hands. The mention of this name will awaken thoughts in the memories of many, of the sad fate of this truly gifted but unfortunate child of genius, far beyond the boundaries of the Palmetto state, in which he was born, and where all that was mortal will quietly repose until the earth and the sea shall give up their treasures, and the dead shall come forth at the command of God. But the genius he gave to the world of poesy and of song, shall endure commensurate with the eons yet to come, and is not circumscribed by latitudes nor climes.

The object of the "little book is to obtain means for placing a memorial stone upon the grave of the poet Henry Timrod. A committee has been appointed consisting of five prominent and distinguished gentlemen of South Carolina, and its chairman, Hon. Hugh L. Thompson, of Columbia, S. C., has kindly consented to act as treasurer, and receive such amount as you may feel able to contribute." All who would like to mark the resting place of poor Harry are respectfully invited to send contributions to Mr. Thompson.

